# A Systematic Review on the Role of Βeta-Blockers in Reducing Cardiac Arrhythmias in Long QT Syndrome Subtypes 1-3

**DOI:** 10.7759/cureus.17632

**Published:** 2021-09-01

**Authors:** Terry R Went, Waleed Sultan, Alisha Sapkota, Hajra Khurshid, Israa A Qureshi, Nasrin Jahan, Anjli Tara, Myat Win, Dwayne A Wiltshire, Amudhan Kannan, Sheila W Ruo, Michael Alfonso

**Affiliations:** 1 Medicine, California Institute of Behavioral Neurosciences & Psychology, Fairfield, USA; 2 Medicine, Beni Suef University Faculty of Medicine, Beni Suef, EGY; 3 Surgery, Halifax Health Medical Center, Daytona Beach, USA; 4 Psychiatry, California Institute of Behavioral Neurosciences & Psychology, Fairfield, USA; 5 Medicine and Psychiatry, California Institute of Behavioral Neurosciences & Psychology, Fairfield, USA; 6 Internal Medicine, California Institute of Behavioral Neurosciences & Psychology, Fairfield, USA; 7 General Medicine, General Surgery, and Emergency Department, Jinnah Postgraduate Medical Centre, Karachi, PAK; 8 Neurosurgery and General Surgery, Liaquat University of Medical and Health Sciences, Karachi, PAK; 9 Neurosurgery and General Surgery, California Institute of Behavioral Neurosciences & Psychology, Fairfield, USA; 10 General Surgery, Nottingham University Hospitals NHS Trust, Nottingham, GBR; 11 General Surgery, California Institute of Behavioral Neurosciences & Psychology, Fairfield, USA; 12 Neurological Surgery Research, Surgical Oncology Research, and General Surgery Research, California Institute of Behavioral Neurosciences & Psychology, Fairfield, USA; 13 Surgical Pharmacology, General Surgery, and Surgery, Jawaharlal Institute of Postgraduate Medical Education and Research, Puducherry, IND; 14 General Surgery Research, California Institute of Behavioral Neurosciences & Psychology, Fairfield, USA; 15 Medicine, Universidad del Rosario, Bogota, COL

**Keywords:** long qt syndrome, beta blocker, arrhythmia, sudden cardiac death, nadolol, propanolol, atenolol

## Abstract

Long QT syndrome (LQTS) is one of the most common inherited cardiac channelopathies with a prevalence of 1:2000. The condition can be congenital or acquired with 15 recognized genotypes; the most common subtypes are LQTS 1, 2, and 3 making up to 85%-90% of the cases. LQTS is characterized by delayed ventricular cardiomyocyte repolarization manifesting on the surface electrocardiogram (EKG) by a prolonged corrected QT (QTc) interval. The mainstay of treatment for this condition involves in part or combination medical therapy via β-blockers as first-line (or other anti-arrhythmic), left cardiac sympathectomy, or implantable cardiac defibrillator placement. Given the high rate of adverse cardiac events (ACE) or sudden cardiac death (SCD) in this population of patients with this disease, this review seeks to highlight the genotype-specific treatment consensus in β-blocker therapy of the most common subtypes.

A database search of PubMed, PMC, and Medline was conducted to ascertain the most recent data in the last five years on the management of LQTS types 1-3 and the role of β-blockers in reducing ACE in these types. The Preferred Reporting Items for Systematic Reviews and Meta-Analyses (PRISMA) guidelines were adhered to in the study selection, and selected studies focused on humans, written in the English Language, and within the last five years of LQTS subtypes 1, 2, and 3.

Eleven relevant studies were selected after considering inclusion criteria, exclusion criteria, and quality appraisal within the last five years, focusing on β-blocker selection directed based on the subtypes of LQTS. Two meta-analyses, one cohort study, and eight reviews provided significant data that non-selective β-blockers unequivocally are of benefit in these LQTS types. Summary of findings suggested nadolol followed by propranolol yields the best results in LQTS 1, while nadolol would yield the best effect in LQTS 2 and 3.

## Introduction and background

Long QT syndrome (LQTS) is both an inherited and acquired condition that is characterized by an abnormal prolongation of the QT-interval on electrocardiogram (EKG) monitoring and action potential duration (APD), which increases the risk of fatal ventricular, arrhythmias, sudden cardiac death (SCD), and milder adverse cardiac eventsACE such as syncope [1-2]. This condition was first officially coined and supported by EKG evidence in 1957 and has since been recognized as one of the most commonly inherited channelopathies/arrhythmia syndromes with a prevalence of 1:2000 [2]. Congenital LQTS has more than 15 genotypes of which the most common genes are LQT1, LQT2, and LQT3 [3]. The primary and most often first reported symptomatic presentation is syncope, and the incidence of sudden cardiac death (SCD) is 1%-3%; though rare, the outcome is significant [3-5].

Brief details of the three most common LQTS subtypes are presented in Table 1, highlighting the causative genes, mutations of said genes, and the affected current channels associated with each syndrome subtype [5].

**Table 1:** Channel mutations in LQTS1, LQTS2, and LQTS3 and the ionic currents affected Ina, Cardiac sodium channel; IKs, slow component of the delayed outward rectifier channel (potassium); IKr, rapid component of the delayed outward rectifier channel (potassium); KCNQ1, potassium voltage-gated channel subfamily Q member 1; HERG, human ether-a-go-go-related gene; SCN5A, sodium voltage-gated channel alpha subunit 5.

LQTS	Proportion of cases	Gene	Channel	Physiological function	Current changes
LQTS1	~50%	KCNQ1	K_V_7.1	I_Ks_: l phase 3 slow activation component of the delayed rectifier potassium current during action potential repolarization	Reduced I_Ks_
LQTS2	~40%	HERG	K_V_11.1	I_Kr_: phase rapid activation component of the delayed rectifier potassium current during action potential repolarization	Reduced I_Kr_
LQTS3	~10%	SCN5A	Na_V_1.5	I_Na_: phase 0 depolarization of the action potential	Increased late current I_Na-L_

The three major LQTS genes depicted above KCNQ1, HERG, and SCN5 account for 49%, 39%, and 10% of LQTS cases as stated by Saadeh et al. [5]. Mutations in each of these genes lead to the clinical presentation of LQTS via prolongation of the action potential duration.APD. The loss of function mutation in KNCQ1 (encoding KV7.1 channel) and HERG (encoding KV11.1 channel) leads to the clinical presentation of LQTS 1 and 2, respectively, while LQTS 3 is found to have a gain-of-function mutation of SCN5A encoding the NaV1.5 channel [6-8].

In LQTS 1, KCNQ1 encodes the a-subunit of the potassium (K+) channel Kv 7.1, whose role is the generation of IKs. IKs, along with IKr, are the two main components of the delayed rectifier IK current that is the main determinant of phase 3 repolarization or action potential generation in ventricular myocytes. In impairment of IKs, in this case, through loss of function mutation, there is a failure of adaptation to sympathetic stimuli and resultant tachycardia or elevated heart rates. The consequence of which is the susceptibility of persons with this mutation to arrhythmias in states that are highly adrenergic for example exercise. This relationship highlights the association between exercise and symptomatic presentations in LQTS type 1 patients [5,6,8].

LQTS 2 is more symptomatic during periods of emotional stress or distress or even auditory stimuli in comparison to LQTS 1 whose symptoms are triggered by exercise, although events secondary to exercise are not uncommon. Despite similarities in potassium channel mutation in LQTS 2, KCNH2 encodes the pore-forming alpha subunit of the cardiac voltage-gated potassium channel Kv11.1 as references in Table 1 and by Saadeh et al. and Etheridge et al. in their respective papers [5,6,9]. The prolongation of action potential evidenced by prolongation of the QTc interval is due to the delay of the conduction of the delayed outward rectifier channel (potassium) (I­kr) by the Kv11.1 channel complex. As the I­kr contributes to phase 3 repolarization, the loss of function mutation indicative of LQTS subtype 2 leads to a reduction in this repolarizing current and thus prolongation of the action potential [9,10].

Finally, LQTS 3 has two distinct differences from its counterparts LQTS 1 and 2. The first difference is the etiology via gain of function mutation as opposed to loss of function mutations, and second, its mutation is on the I­­­Na ­(sodium channel in comparison to IK channel). LQT3 is characterized by bradycardia, prolonged ST segment, and late-onset of the T wave on EKG and is a result of gain-of-function mutations in the SCN5A gene that encodes the a-subunit of the cardiac Na­+ channel NaV 1.5 [5,7]. This sodium channel’s main role is the generation of inward current (INa) that results in the phase 0 depolarization of the cardiac action potential. Mutations that cause LQTS 3 see a Nav 1.5 channel that remains activated and thus prolongs repolarization and APD by extension the QT interval [9]. This is in comparison to the normal action potential sequence of Nav1.5 being inactivated after depolarization and no conducting significant current during the repolarization phase [5-7].

Diagnosis of LQTS is done by clinical symptoms in conjunction with EKG assessment and is best done using the Schwartz Diagnostic Criteria for LQTS, which is highlighted in Table 2 [7].

**Table 2:** Schwartz diagnostic criteria for long QT syndrome (2011) *In the absence of medications or disorders known to affect these electrocardiographic features. ^†^QTc calculated by Bazett formula where QTc = QT/√RR. ^‡^Mutually exclusive. ^§^Resting heart rate below the second percentile for age. ^||^The same family member cannot be counted in A and B. If the score is ≤1 point, low probability of LQTS; 1.5–3 points: intermediate probability of LQTS; ≥3.5 points: high probability. LQTS, Long QT syndrome.

ECG Findings (QTc)*		Points
A	QTc, † ms	
	≥ 480	3
	460 to 479	2
	450 to 459 (in males)	1
B	QTc 4^th^ minute of recovery from exercise stress	1
C	Torsades de pointes^‡^	2
D	T wave alternans	1
E	Notched T wave in three leads	1
F	Low heart rate for age^§^	0.5
Clinical History		Points
A	Syncope^‡^	
	With stress	2
	Without stress	1
B	Congenital deafness	0.5
Family history		Points
	Family members with definite LQTS^||^	1
	Unexplained sudden cardiac death below age 30 among immediate family members^||^	0.5

In treating this arrhythmia syndrome, the mainstay of medical management is supported by β-blocker therapy [1,2,6]. Additional treatment modalities include medical via mexiletine, ranolazine, and/or flecainide, as well as procedural, via left cardiac sympathetic denervation and implantable cardiac defibrillator (ICD) [11-13]. The increased late sodium currents (INa-L) in LQTS and LQTS 3 can result in bradycardia-dependent QT prolongation, given that mexiletine is an inhibitor of INa-L [14,15]. Mexiletine has an increased role and benefits in treating and managing patients in LQTS 3, in which β-blocker therapy may be a contraindication (patients with both significant bradycardia and QT prolongation) that may be further worsened by the anti-adrenergic (bradycardic) effect of β-blockers on lowering heart rate even further [16]. ICDs have long been the mainstay of management of severe LQTS regardless of subtype. Prior to recent times, ICDs had been the main treatment modality for LQTS 3, but other treatment options are still proposed [11].

Some studies have shown that although β-blocker therapy is effective in LQTS, different subtypes of LQTS show differing responses to this therapy and other modalities such as mexiletine and ranolazine have a better response in other types, like in case of LQTS type 3 as compared to LQTS type 1 or 2 [8-10,16]. The current data suggests varying thought processes on the role of β-blockers and specific β-blockers in different LQTS subtypes.

The lack of randomized clinical trials has been a consistent limitation in research, and several studies had conflicting data results on preferred treatment modalities especially where LQTS 3 is concerned. This study seeks to build on the data in the previous years and review the most recent available studies in the last five years and determine both the effects of β-blockers on the more common LQTS subtypes (1-3) and the effectiveness of β-blocker therapy in reducing fatal and non-fatal arrhythmias among these subtypes.

## Review

Method

Data Sources and Search Criteria

The guidelines for systematic review according to the Preferred Reporting Items for Systematic Reviews and Meta-Analyses (PRISMA) statement were adhered to in preparing this systematic review. Databases used for study retrieval were PubMed, PMC, and Medline, which were searched to ascertain the available data within the last five years in the use and effectiveness of β-blockers in LQTS in reducing the risk of cardiac arrhythmias in LQTS depending on the genotype of the more common LQTS subtypes (1, 2, and 3). The relevant studies were determined from the search by the following criteria.

Definition and Study Eligibility (Inclusion/Exclusion Criteria)

The research question identified assessing the role of β-blockers in reducing adverse cardiac eventsACE in congenital LQTS types 1, 2, and 3. Cardiac events (CEs) included both non-fatal like syncope, aborted cardiac arrest (ACA), and fatal such as sudden cardiac death (SCD). The inclusion criteria were primarily studies within the last five years (with some exceptions based on high relevance), human studies, publications in the English language, and peer-reviewed literature; types of the studies included clinical and review studies, studies involving patients with congenital LQTS on β-blockers, and studies involving patients with congenital LQTS who developed arrhythmias. Exclusion criteria included studies of more than five (5) years of age, studies of acquired LQTS and low relevance, LQTS not of types 1-3, animal studies, non-English studies.

Search Strategy

A thorough advanced search strategy was undertaken on April 20, 2021, and the relevant studies were identified using a combination of generic keywords and Medical Subject Headings (MeSH) search terms. The initial broad search of the databases using the terms “Long QT syndrome” AND “β-blockers” AND “arrhythmias” produced a total of 4592 studies of which 12 duplicates were identified.

The study focused on studies within the last five years (2016-2020). Initial screening showed 428 relevant studies on PubMed, PMC, and Medline. The inclusion/exclusion criteria highlighted above were applied to 428 studies, and the results were narrowed to 66. These were primarily studies from 2016 to 2020. Mendeley Reference Manager was utilized to organize the articles used in the review. After application of the inclusion/exclusion criteria, the results were further narrowed to 15, pending quality assessment by TW and WS. After quality appraisal and final review, a final tally of 11 studies, two meta-analyses, one cohort study, and eight literature reviews were utilized for this review.

**Figure 1:**
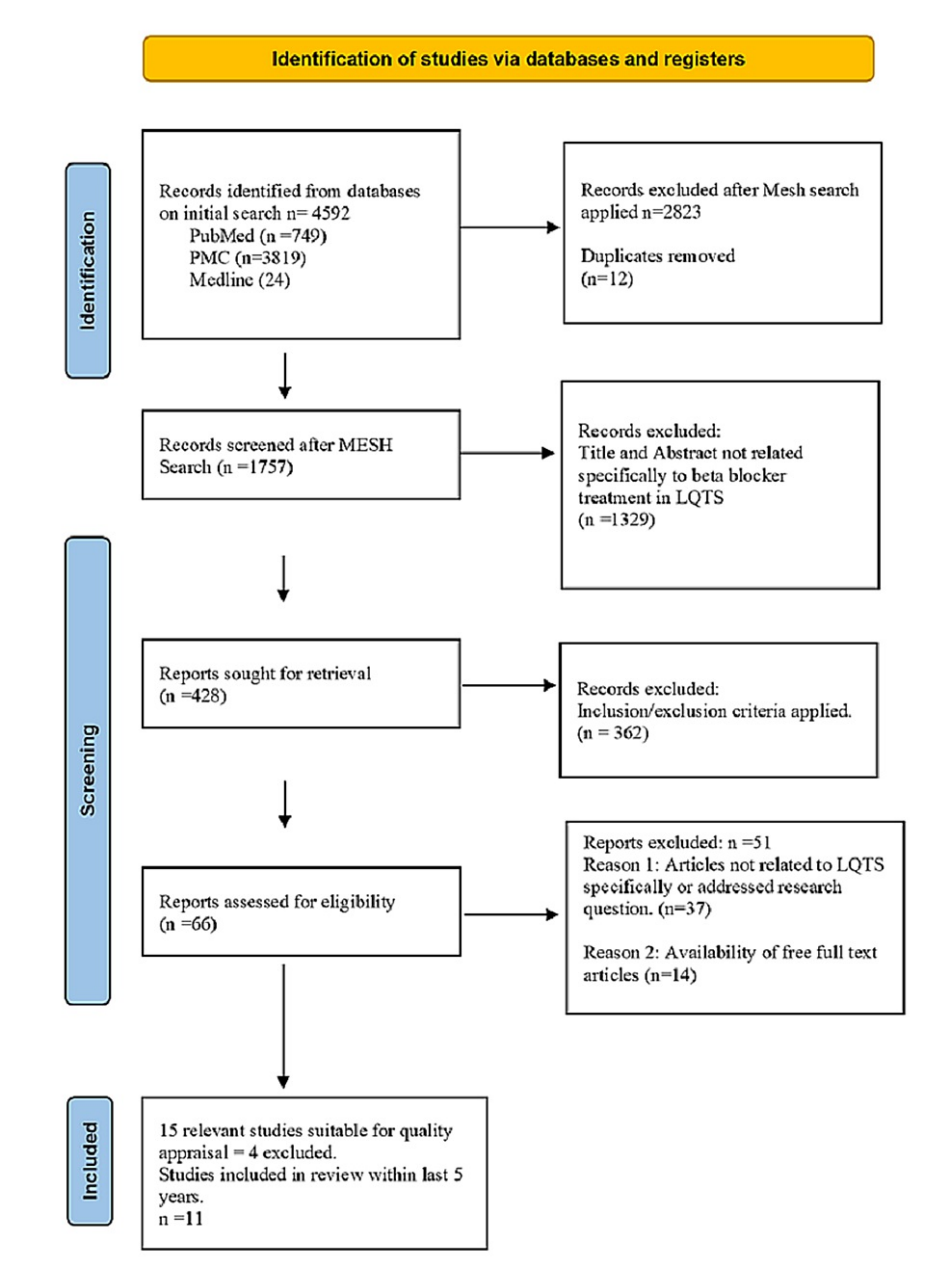
PRISMA flow diagram (2020) PRISMA, Preferred Reported Items for Systematic Review and Meta-analysis [17]; n, number.

The screening was performed by two investigators/authors (TW and WS) and screened all the papers by title and abstract based on the criteria of the study. The PRISMA statement flow diagram in Figure 1 reflects the process of article selection and filtering with inclusion/exclusion criteria for this review [17].

Quality Assessment

The quality of the studies selected was assessed by the first and second authors independently using the Newcastle-Ottawa Quality Assessment Scale for the registry-based cohort studies by Wilde et al. and the AMSTAR assessment tool for meta-analysis and SANRA checklist for the remaining eight literature reviews. Fifteen studies were assessed for quality appraisal, and 11 studies fulfilled the quality requirements. Disagreements were settled by MA as an arbiter for accuracy and precision [18-20].

The following PRISMA flow diagram (Figure 1) highlights the aforementioned steps.

Results

Given the relatively limited studies on LQTS within the last five years to ascertain a broad view of the data, we were able to identify 4592 papers in the initial database search on the topic, which was then narrowed to 1757 after MeSH criteria were applied. Mendeley Citation Manager was used to assess the studies, and 12 duplicates were identified. After the first level of eligibility screening of the 1757 articles for the title and abstract relevance, 428 studies were sought for retrieval. Inclusion and exclusion criteria were applied to further narrow the results to 66 and obtain more current data within the last five years. Fifteen studies were selected and assessed for quality appraisal, which led to the selection of 11 studies. These 11 articles included for the review were published in peer-reviewed journals from 2016 to 2020 and were relevant to the research question.

Among the studies reviewed, a detailed meta-analysis was provided by Ahn et al. and Han et al. on the risk reduction of all CEs and ACE when β-blockers are part of management. Ahn et al. supported the use and role of nadolol in risk reduction in LQT1 and LQT2 [1]. Han et al. supported this; they focused heavily on the significant reduction in ACE in LQT1 and LQT2 by β-blockers, and the role of nadolol was seen as being ideal in both types, with atenolol a possible substitute in LQT1 [2]. Saadeh et al. 2020 confirmed the role of β-blockers in LQTS as a whole and emphasized the efficacy and role of nadolol in LQTS 3, where previously β-blockers were thought to be contraindicated [5]. Winbo et al. despite focusing on sympathetically triggered arrhythmia syndromes concluded that the provided non-selective β-blockers are more efficient in reducing arrhythmia risk than beta 1 selective such as metoprolol or bisoprolol [21]. Etheridge et al., Ackerman et al., and Ortmans et al. had similar conclusions as Saadeh et al. supporting non-selective β-blockers such as nadolol and propranolol as first-line treatment options [5,6,13,22]. Wilde et al. in 2016 provided the largest multicenter cohort study on LQT3, and their conclusions led to the recommendation of nadolol as the first line for this subtype [14]. Steinberg purported the need for an arrhythmia clinic for risk stratifying and individualized management of cases of LQTs [23].

They provided unanimity that nadolol is the most effective β-blocker in LQTS and should be used as first-line treatment for secondary prevention and primary prevention in high-risk individuals [23]. Finally, the two papers by Waddell-Smith et al. (2016) and Waddell-Smith et al. (2015) looked at the risk reduction provided by β-blockers in females and children, respectively, and saw benefits in each study. In their 2016 study, the overall reduction of risk of SCD by β-blockers (nadolol) and a superior protective effect were noted versus other types of β-blockers. In children studied in their 2015 paper, nadolol remained the first line with controlled-release propranolol as an alternative [24,25].

Discussion

LQTS is a congenital or acquired arrhythmogenic disorder characterized by repolarization abnormalities (elongation of the QTc interval > 470 ms). LQTS is characterized by elongated ventricular action potential duration (APD) with a propensity to cause life-threatening CEs [5]. The first manifestation of this syndrome may be SCD/fatal ventricular arrhythmia that highlights the significance and importance of appropriate genotype-specific therapy [13,26,27].

β-Blockers and LQTS

β-blockers as a class have long been recognized as the primary/first-line treatment of choice for LQTS as their anti-adrenergic effect (β-blockade) has been critical in reducing ACE (adverse cardiac events) among the three major genotypes of LQTS, which are LQT1, LQT2, and LQT3 (all three account for more than 80%~90% of the mutations identified in congenital LQTS) [1,27,28]. β-blockers by their name act on and block the signaling of the β-adrenergic receptors on the myocardium, primarily β1 adrenergic receptors (β1-AR) and to a lesser extent on β2-adrenergic receptors (β2-AR) in order to reduce signaling that would otherwise facilitate both chronotropic primarily but by extension ionotropic activity. β-blockers by their anti-sympathomimetic activity decrease the etiology of cardiac arrhythmias/ACE in LQTS as sympathetic activity increases the risk of these events [5,27,28]. Studies by Ahn et al., Winbo et al., and even Gemma et al. found patients on EKG monitoring during exercise, and recovery support showed that β-blockade reduced the QT interval [1,21]. Ortmans et al. referenced statistics of untreated LQTS mortality from SCD to be between 0.3% and 0.9% per year and other ACEs (not SCD), approximately 10% from birth to 40 years of age in asymptomatic carriers of pathogenic mutations [22]. Notably and understandably, no two β-blockers are the same and have varying pharmacodynamic and kinetic effects that show their efficacy in varying subtypes [28,29]. Table 3 presents study characteristics.

**Table 3:** Studies selected for review and their respective findings LQTS, Long QT syndrome; ACEs, adverse cardiac events.

No.	Study	Type of Study	LQT Subtype Studied	Findings
1	Han et al., 2020 [2]	Meta-analysis	LQTS1-3	This study of β-blocker treatments showed a significant risk reduction for ACEs in LQTS patients and showed that nadolol provided a strong risk reduction for ACEs in LQTS patients (HR 0.42, 95% CI 0.25–0.70). Nadolol’s efficacy was more pronounced in LQT2 than in LQT1 patients; the direct comparison between the groups revealed that this was statistically significant (LQT2 vs. LQT1, HR 0.53, 95% CI 0.27–1.04). For LQT1 patients, nadolol ranked first with higher efficacy for reducing CEs risk, second-ranked in efficacy was atenolol as an effective treatment of the four β-blockers for patients with LQT1. Propranolol was ranked third and followed by metoprolol fourth. In LQT2 patients, nadolol was unequivocally verified to be the first-line therapy with minimal risk of CEs. Notably, a significant reduction in ACE was not seen in LQT3 (HR 0.63, 95% CI 0.36–1.10, p = 0.43). Of note, the incidence of ACE is higher in this subtype.
2	Saadeh et al., 2020 [5]	Review	LQTS 1, LQTS 2, LQTS 3.	Combined the most recent data at the time of both animal and experimental studies to sought to ascertain the efficacy of β-blockers in LQTS management. They looked at both the adrenergic stimulation and blockade and its symptomatic outcome in these patients. Their findings concluded that the anti-adrenergic effect of β-blockers was not consistent across the board. The anti-arrhythmic efficacy of specific β-blockers determined their effectiveness depending on the specific genotype of Long QT syndrome being treated. In LQTS 1 and LQTS 2, the role of β-blockers is clear, and with LQTS 3, the main effective β-blocker recommendation was nadolol.
3	Etheridge et al., 2019 [6]	Review	LQTS 3	Provided an update on the new clinical and genetic aspects of long QT syndrome (LQTS) especially in a controversial treatment of LQTS 3 with β-blockers, especially where treatment with nadolol was indicated as an effective alternative.
4	Winbo et al., 2019 [21]	Review	LQTS 1-3	Reviewed not only LQTS but sympathetically triggered inherited arrhythmia syndromes such as catecholaminergic polymorphic ventricular tachycardia (CPVT) and the resultant cardiac death in young patients with normal cardiac anatomy. They focused on the role of neuromodulator methods in treating symptoms, and non-selective β-blockers (nadolol and propranolol) are more efficient in reducing arrhythmia risk than beta 1 selective such as metoprolol or bisoprolol.
5	Ortmans et al., 2019 [22]	Review	LQTS 1, LQTS 2, LQTS 3	These are highly effective, reducing the risk of cardiac events by approximately 95% in LQT1, 75% in LQT2, and 80% in LQT3. Long-acting β-blockers such as nadolol or propranolol should be preferred over shorter-acting agents such as metoprolol.
6	Steinberg 2018 [23]	Review	LQTS 1-3	Purported the need for an arrhythmia clinic for risk stratifying and individualized management of cases of LQTs. There is unanimity that nadolol is the most effective β-blocker in LQT and should be used as first-line treatment for secondary prevention and primary prevention in high-risk individuals. Bisoprolol may be an attractive alternative for asymptomatic, low-risk individuals with LQTS.
7	Ahn et al., 2017 [1]	Meta-analysis	LQT 1-3	Highlights that the use of β-blockers was associated with a significant risk reduction of all cardiac events (HR 0.49, p < 0.001 in Cohort; RR 0.39, p < 0.001 in ITS) and adverse cardiac events (ACE or SCD) (HR 0.47, p < 0.001 in Cohort). Nadolol showed significant risk reduction in both LQT1 and LQT2 (HR 0.47 and 0.27, respectively) when compared to all non-selective β-blockers and selective, whereas atenolol and propranolol had a modest effect in decreasing the risk only in LQT1 (HR 0.36 and 0.46, respectively). Of note, metoprolol showed no significant reduction in either genotype.
8	Ackerman et al., 2017 [13]	Review	LQTS 1-3	Provided a consensus in the use of nadolol as the first line in treatment of LQTS and a possible alternative being propranolol with no specific reference to genotype.
9	Wilde et al., 2016 [14]	Cohort study	LQTS 3	Clinical aspects of type 3 long-QT syndrome looked at 406 LQT3 patients with 51 sodium channel mutations (391 patients were event-free during the first year of life). The findings showed prolonged QTc, and syncope predisposes patients with LQT3 to life-threatening CEs (cardiac events). However, β-blocker therapy reduces this risk in females; efficacy in males was inconclusive, given limited events. β-blocker therapy reduces the risk for cardiac events in patients with LQT1 by >95% and in patients with LQT2 by 70%-80%. In contrast, the early genotype-phenotype studies showed no demonstrable β-blocker efficacy for LQT3.
10	Waddell-Smith et al., 2016 [24]	Review	LQTS 1-3	β-blockade initiation was done for patients that were symptomatic or with a QTc > 470 ms. The overall reduction of risk of sudden cardiac death by β-blockers in high-risk subjects is 67% in LQT1 males and 71% in LQT2 females. In long QT 3, a suggested risk reduction of 33.33% was highlighted in women > men. Nadolol may have a superior protective effect to other β-blockers in long QT type 2 and may be the most effective at preventing first cardiac events in LQT1 and LQT2, and propranolol may be least effective in those with prior cardiac events. Notable in LQT3, medical intervention was found to be more beneficial in combination with ICD placement.
11	Waddell-Smith et al., 2015 [25]	Review	LQTS 1-3	Looked at treatment in children and though rare if β-blockers are given, a long-acting, single daily preparation encourages/optimizes adherence, and nadolol is the first line with an alternative being controlled-release propranolol.

Role of β-Blocker in LQTS 1

LQT 1 is represented by a KCNQ1 mutation (encoding for the cardiac potassium channels) that leads to IKs current reduction, the sequelae of which causes prolongation of the QT interval; this means a delay in repolarization of the ventricles. As mentioned, sympathetic stimuli in the form of exertional activity or exercise are a potent trigger that can further exacerbate this state. β-blockers by their nature reduce sympathetic activity and reduce QT prolongation and by extension the negative side effects of syncope and possible ACE [6,27,28]. Saadeh et al. in their review provided convincing evidence to the role of β-blockers in LQTS syndrome as a definitive management option and addressed the previous concerns for the three main subtypes. In subtype 1, the β-blockers of choice for reducing ACE have been non-selective β-blockers such as nadolol and propranolol, which have shown significant reductions in CEs when compared to non-treated patients [5]. This is supported by retrospective cohort studies that were referenced by Saadeh et al. in past years, which showed a 79% reduction in the first CE by β-blockers [5].

Role of β-Blocker in LQTS 2

LQT2 is due to loss of function mutations in the KCNH2 gene (encoding for the cardiac potassium channels) that results in decreased IKr current propagation like LQTS 1 in prolongation of the QT interval and the resulting sequelae of syncope and ACEs. The consensus has been maintained in prior studies that β-blockers have reduced efficacy as compared to their role in LQTS 1, given that the role of sympathetic stimulation seen in LQT1 has a greater effect than in LQT2 [7]. This difference in effect has been purported to have relation to the reduced role of sympathetic triggers such as exercise, due to the presence of alpha1A adrenoreceptor-mediated IKr reduction, and lower incidences of ACEs in young patient cohorts and their etiology from sympathetic triggers and more so from triggers such as emotional or auditory arousal [1,6,7]. This is further supported by retrospective cohort studies referenced by Saadeh et al. in past years, which showed a 79% reduction in the first CE by β-blockers in LQT1 versus 63% reduction in LQT2. Nonetheless, despite the relative increased ACEs in LQT2 while on β-blocker therapy comparatively to LQT1 patients, it remains the first-line medical therapy in this condition in reducing both non-fatal and fatal ACEs; this is significant especially in conjunction with mexiletine [25,26].

Role of β-Blocker in LQTS 3

LQTS 3 is the result of gain-of-function mutations in the SCN5A-encoded Nav1.5 sodium channel. Although a major LQTS subtype with types 1 and 2, LQT3 comprises just about ≈5% to 10% of patients with LQTS, Wilde et al. provided one of the most current and detailed backgrounds in LQTS 3, and they explained in detail the significant difference of this phenotype from the other types 1 and 2, which is in the sodium channel mutation versus the potassium channel mutation [14]. The more significant clinical difference that affects the response to treatment with β-blockers is that LQTS symptoms frequently occur at rest with a limited role of adrenergic/sympathetic triggers or emotions as with types 1 and 2, respectively [4,14]. In comparison with patients with LQT1 and LQT2, patients with LQT3 have a more severe clinical presentation with more lethal first events and lower resting bradycardia. To that end, the role of β-blockers that tend to further lower the heart rate was previously thought to be proarrhythmic [5,12,14].

The medical management of LQTS 3 has long been an outlier in LQTS treatment especially when it comes to the use of β-blockers, which was not quite clear, with some studies showing contradictory results. β-blockers were even thought to worsen this condition as compared to the improvement in the other types of LQTS [3-5]. The role of β-blockers in LQTS 3 has remained a point of contention in previous years, given the proarrhythmic nature of β-blockers in extreme bradycardia (further heart rate reduction), which has understandably led to caution in their use. There was limited and conflicting data in studies in the early twenty-first century; however, within the last five years, in particular, several studies have provided recommendations on effective management. Wilde et al. in their 2016 multicenter cohort study about LQTS 3 refuted the most well-known perceptions on LQTS 3 and the long-held belief in the proarrhythmic nature of β-blockers in this condition. Wilde et al. reviewed the cases of 406 LQT3 patients that had statistically significant findings on the role of β-blockers in reducing CEs in this subset of patients. Their findings and subsequent conclusions showed that β-blocker therapy is protective and significantly reduces CEs, especially in the female population (p-value of 0.014, HR 0.170) [14]. The main limitation was that their recommendation was geared primarily toward female patients, given limited male patient data. These findings nonetheless were also supported by Saadeh et al., whose recommendations support the use of nadolol in patients with LQTS 3 [15,17]. In addition, data provided by Ahn et al., Etheridge et al., Waddell et al., and Adamos et al. also supported the role of β-blockers, nadolol, for the management of LQTS 3, whereas the previous treatment options were mexiletine, ICD, or left sympathetic denervation [1,4,24,28,29].

Table 4 highlights/summarizes the pathophysiology, clinical presentation, and management options and response in LQTS 1, 2, and 3.

**Table 4:** LQTS subtypes, genes, triggers, and management Adapted from Saadeh et al. and Waddel et al. [5,24]. Ina, Cardiac sodium channel; IKs, slow component of the delayed outward rectifier channel (potassium); IKr, rapid component of the delayed outward rectifier channel (potassium). Response to treatment: Strong***, Moderate**, Fair*.

Pathophysiology	LQTS 1	LQTS 2	LQTS 3
Gene	KCNQ1	KCNH2	SCN5A
Ion channel	Reduced I_Ks_	Reduced I_Kr_	Increased late I_Na_
Protein	Potassium (Kv 7.1)	Potassium (Kv 11.1)	Sodium (Nav 1.5)
Clinical presentation	LQTS 1	LQTS 2	LQTS 3
Arrhythmia triggers	Sympathetic stimuli (exercise, stress), emotion	Emotion and stress, startled, arousal from sleep	Sleep/rest
ECG findings	Broad-based T wave	Low amplitude, bifid T wave	Long ST segment (isoelectric)
QT response to exercise	Shortening of QTc and prolongation during exercise	Exaggerated QT hysteresis. However, normal during exercise.	Supernormal QT shortening
Response to treatment	LQTS 1	LQTS 2	LQTS 3
Β-blockers	***	***	variable
Mexiletine	*	*	**
Exercise restriction	***	**	variable
Left cervical sympathectomy	**	**	**

The respective meta-analyses by Ahn et al. (2017) and Han et al. (2020) provided the strongest evidence on the role and effectiveness of β-blockers according to LQT genotype, especially in conjunction with the reviews and cohort studies on the topic. The results of the analysis posited that overall β-blocker therapy was significantly associated with decreased risk for CEs (syncope, ACA, or SCD) [1,2]. Notably, this effect showed a disparity among β-blocker types and LQTS genotype, and the overall effect was more prominent in reducing life-threatening, serious CEs (ACA or SCD) as compared to all events like syncope [1]. The data from these meta-analyses’ provided significant evidence in the role of β-blockers in preventing ACE in all three subtypes, and Han et al. made specific reference to the role of nadolol in LQT2 as well as LQT1 [2]. When placed in the perspective of the respective studies of Waddell-Smith et al., Saadeh et al., Ortman et al., and Winbo et al., nadolol appears to be the ideal potential β-blocker of choice in all LQT subtypes, and atenolol followed by propranolol offers viable alternatives in types 1 [1,2,5,24].

Study limitations

The number of studies in the review reflected limited papers published in the last five years especially as it relates to cohort studies. An additional limitation to this study was the inability to access all the full-text papers available within the criteria outlined. Furthermore, although there is some consensus on the role of β-blockers in treating LQTS types 1 and 2, the role of nadolol in treating LQTS subtypes 3 still requires further in-depth study. Most analysis of LQTS has been limited to retrospective analysis due to the significant clinical role of β-blockers in treating the condition and ethical considerations of a randomized clinical trial potentially in withholding lifesaving treatment in the placebo group. A possible solution to this would be utilizing a significantly larger sample size and assessing the variations in CEs occurrence, type, and duration based on the LQT subtype and beta-blocker of choice selected.

## Conclusions

In regard to LQTS, this systematic review provided a look at the latest updates in the research on the effects of β-blockers on reducing fatal ventricular arrhythmias and the variance based on genotypes of LQTS types 1, 2, and 3. The consistency in the data reflected that a genotype or subtype-based approach to β-blocker therapy in treating LQTS remains an important point of discussion. β-blockers such as atenolol or propranolol were effective treatment modalities for the subtypes 1 and 2, and propranolol appears to have some superiority in comparison with other β-blockers; however, nadolol remains the most effective treatment as a β-blocker in all LQTS subtypes with significant effects in LQT2 and 3. Ultimately, randomized clinical trials or larger prospective studies may be required to better elucidate the roles of specific β-blockers, especially nadolol, and propranolol in the LQTS genotypes to provide targeted therapies.
